# Classification of COVID-19 and Influenza Patients Using Deep Learning

**DOI:** 10.1155/2022/8549707

**Published:** 2022-02-28

**Authors:** Muhammad Aftab, Rashid Amin, Deepika Koundal, Hamza Aldabbas, Bader Alouffi, Zeshan Iqbal

**Affiliations:** ^1^Department of Computer Science, University of Engineering and Technology, Taxila, Pakistan; ^2^Department of Systemics, School of Computer Science, University of Petroleum & Energy Studies, Dehradun, India; ^3^Prince Abdullah Bin Ghazi Faculty of Information and Communication Technology, Al-Balqa Applied University, Al-Salt, Jordan; ^4^Department of Computer Science, College of Computers and Information Technology, Taif University, P.O. Box 11099, Taif 21944, Saudi Arabia

## Abstract

Coronavirus (COVID-19) is a deadly virus that initially starts with flu-like symptoms. COVID-19 emerged in China and quickly spread around the globe, resulting in the coronavirus epidemic of 2019–22. As this virus is very similar to influenza in its early stages, its accurate detection is challenging. Several techniques for detecting the virus in its early stages are being developed. Deep learning techniques are a handy tool for detecting various diseases. For the classification of COVID-19 and influenza, we proposed tailored deep learning models. A publicly available dataset of X-ray images was used to develop proposed models. According to test results, deep learning models can accurately diagnose normal, influenza, and COVID-19 cases. Our proposed long short-term memory (LSTM) technique outperformed the CNN model in the evaluation phase on chest X-ray images, achieving 98% accuracy.

## 1. Introduction

A novel coronavirus known as COVID-19 has been a global health threat since 2019. This virus produces severe acute respiratory syndrome. According to a study conducted by John Hopkins University, there are 22,688,934 confirmed cases and 793,773 fatalities worldwide as of August 2020 [1]. The United States, Brazil, and India have the most reported cases and deaths in 2020 [2]. After June 2020, the virus rapidly spread around the globe. COVID-19 has three stages: mild, moderate, and critical [3]. Symptoms of the mild stage are dry cough, fever, and tiredness. The intermediate stage symptoms are deep cough, muscle pain, and chills with repeated shaking. The patient with moderate stage has higher fever level as compared to mild stage. At the critical level, 50% of the lungs are also infected; discomfort, and bullish face. Multiorgan and respiratory system failure also occur in critical conditions that may result in death. The risk of acute or critical sickness is more than 50% among those over the age of 40, according to statistics from the National Center for Health Statistics (NCHS). Cancer, chronic renal disease, cardiovascular disease, recurrent respiratory disease, type 2 diabetes, and sickle cell disease are linked to an increased risk of serious illness in those with COVID-19 in older age. Clinical trials for vaccination or possible therapy are taking place all around the world.

The first case of the epidemic was detected at the end of 2019 in Wuhan, China [4, 5]. The virus was then spread to the capital of Hubei Province in China and many other countries across the world. Effective and reliable screening and prompt medical care for infected individuals are crucial for the prevention of COVID-19. Around the world, reverse transcription-polymerase chain reaction (RT-PCR) detects COVID-19. The (RT-PCR) process, on the other hand, takes a long time to complete [6]. Therefore, during the epidemic's initial stages of spread, bad RT-PCR accuracy may not be appropriate in many countries. CT and X-ray imaging of the chest may be employed instead of the RT-PCR technique due to a lack of sufficient assays for accurate diagnosis and varying degrees of disease evaluation. UNICEF is a global organisation that strives to save lives, defends rights, and assists children reach their full potential in over 190 nations and territories. Along with the coronavirus, misinformation has spread, leading to discrimination and stigma. UNICEF is working with health experts to find the precautions and improving immune system of children. The global programs and activities of UNICEF are based on extensive research and careful evaluation of the needs of children.

COVID-19 has had a substantial impact on low and middle-income economies, as well as gender disparities in employment, according to the Bill and Melinda Gates Foundation. According to the International Labor Organization, women should have 13 million fewer employments after the outbreak than in 2019. On the other hand, men's jobs are likely to revert to levels seen in 2019. As the disease spreads swiftly into emerging nations, the World Bank Group is working hard to assist clients. The bank has contributed over $157 billion to tackle the pandemic's effects from the commencement of the COVID-19 issue. It comprises roughly $50 billion in IDA resources on grants and exceptionally concessional conditions from April 2020 to June 2021. An influenza-like virus is to blame for the COVID-19 epidemic (SARS-CoV-2). Malnutrition has been associated with a worse viral infection outcome (both under and overnutrition) [7]. The influenza pandemic of 2009 was brought on by a virus known as “Influenza A Virus” (IAV) H1N1.

This virus increased the risk of severe illness. An RNA virus from the Orthomyxoviridae family causes influenza. Two major internal proteins separate the virus into three forms (A, B, and C) [8, 9]. The flu is a disease that is underestimated, maybe because it is a common chronic disease that we all experience and quickly recover from on our own [10]. Respiratory infections killed 4.1 million individuals in 1999, according to the WHO [11]. As a result, they are the most dangerous type of infectious disease. Influenza is to cause of many of these deaths. It is important to realize that flu primarily kills or causes the deaths of older people, resulting in fewer years of life lost than these high fatality rates would suggest. On the other hand, flu is flu pandemic that can cause significant deaths in people of all ages, and they are a concern to persons of all ages with various chronic medical conditions [12].

A novel approach for detecting COVID-19 was proposed using publicly available chest X-ray and CT images by [13], which compared various DL feature extraction methods to find the most accurate features. Cabitza et al. [14] employed machine learning using blood tests data to detect COVID-19 disease. The focus of this work is to promote early detection and treatment of the deadly virus. Ismael and Sengur presented the pertained convolutional neural network (CNN) and created the CNN model after fine-tuning and automated feature extraction [15]. The deep features were classified using the SVM model with various kernel functions. Chen [16] developed a CNN technique that was trained and tested using a publicly available X-ray dataset for tertiary and normal categorization. The proposed model has an 85% tertiary classification accuracy. This article aims to build deep learning algorithms (CNN and LSTM) for detecting normal, influenza, and COVID-19 cases from X-ray images.

This article used deep learning techniques on X-ray images of COVID-19 and influenza for classification and detection. Influenza can also be detected by chest X-ray images using proposed models like COVID-19. In no way can this exploratory study be used to replace medical advice. The rest of the article is set out as follows.  Section 2 presents related work, while Section 3 introduces the problem statement.  Section 4 discusses symptoms of COVID-19 and influenza. In contrast, Section 5 provides a background review of the deep learning approaches and presents the proposed technique. Finally, Section 6 presents the experiments and results, while Section 7 concludes the article.

## 2. Related Work

The various articles have been published during the last two years to detect COVID-19 using different techniques. Some research studies will be discussed in this section. To classify COVID-19 and pneumonia, Nath et al. [1] proposed a DL-based novel approach. The proposed CNN architecture improves X-ray and CT image classification accuracy 99.68% and 71.81% compared to other traditional models. Henzel et al. [17] presented a methodology for detecting and classifying COVID-19. During the assessment of COVID-19 individuals, the suggested technique reveals a study on classifiers modified to acquire an assumed adequate threshold of negative predictive values. Brunese et al. [18] presented the deep learning approach for COVID-19 detection from X-ray images. The proposed approach comprises three stages: the first detect pneumonia from a chest X-ray and the second detects COVID-19 in a chest X-ray. COVID-19 is presently in the second stage of its separation from pneumonia. The third stage is to find the spots in the X-ray that indicate COVID-19 presence. The proposed system has a 97% accuracy rate. For a descendant of the 1918 pandemic strain of the human influenza virus, Goto et al. [19] identified a novel process of HA cleavage. The authors show that neuraminidase, the second most abundant protein on the virion surface, binds and sequesters plasminogen, resulting in higher local concentrations of this ubiquitous protease precursor and, as a result, increased HA cleavage.

The MSDII FFNN model was proposed by Khan et al. [20], in which cloud data are processed, shared, and updated on a regular area-wide basis. In addition, the proposed model predicts which influenza subtypes are most likely to trigger a pandemic. This information can be used to halt disease transmission and minimize damage in a specific area. It would even make it easier for the government to deal with the pandemic. Yin et al. [21] suggested a novel technique for human influenza vaccine prediction. According to the proposed process, the expected findings indicate close matches between the recommended vaccination strains and the circulating strain in various seasons. The proposed model can be employed as a generic solution for other influenza subtypes, such as H1N1 and influenza B, and improve vaccine selection in H3N2. Meanwhile, it offers a fresh viewpoint that allows for the discovery of alterations, such as those caused by viral evolution.

A model was proposed [22] with numerous characteristics (RT-PCR, CT characteristics, and blood test data). The proposed algorithm (random forest algorithm) achieves 92% accuracy in the training phase, while it achieves 96% accuracy in the testing phase. Their suggested model has an F1-score value of 80%, while the Mathews correlation coefficient (MCC) is 76%. A survey was presented by Luzi et al. [23]. The main objective is waiting for a COVID-19 vaccine to be created, isolating positive cases, and social distancing. However, a recent influenza pandemic study believes the following methods improve immune response: the first is to lose weight by following a mild calorie limit. Second, treat obesity-related diabetes with drugs, use AMPK and PPAR gamma activators, and engage in mild-to-moderate physical activity. To anticipate and analyze COVID-19, Taj et al. [24] suggested a series of ML and DL models. Their suggested prediction model effectively makes critical decisions that result in a faster response and situation control. Hammoudi et al. [25] proposed a unique approach for diagnosing pneumonia cases with 70% accuracy at COVID-19. This study investigates deep learning algorithms for autonomously processing chest X-ray images providing doctors with more reliable tools for screening COVID-19 patients and detecting confirmed instances. The study concludes that virulent pneumonia epidemics are likely to be COVID-19 infections during a COVID-19 outbreak.  Table 1 presents the comparison of existing studies related to the proposed work.

## 3. Problem Statement

COVID-19 and influenza are two different diseases, but the symptoms of these two diseases are almost the same. Due to various similar symptoms, the classification of COVID-19 and influenzas is a leading problem in current days. Classification of COVID-19 and influenza is crucial because if patients cannot take treatment for influenza, mostly patients recover by nature. The virus of influenza spreads from an infected patient to other people, but that is not fatal. But in COVID-19, if the virus cannot be detected earlier, it may cause deaths, as the virus of COVID-19 can be transferred to others. If the virus in the carrier cannot be detected earlier, the virus attacks the patient's lungs. Therefore, in COVID-19, it is essential to isolate the affected person since the virus might be transmitted to others. We employ DL techniques for COVID-19 and influenza classification to resolve this issue. The proposed scheme is helpful in classifying COVID-19 and influenza X-ray images of the chest of patients. The suggested method makes it much easier to identify COVID-19 and influenza.

**Figure 1:**
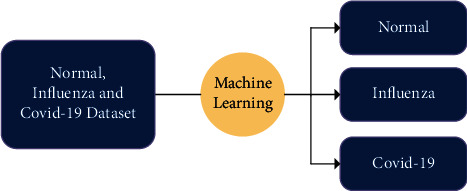
Problem diagram.

As shown in Figure 1, we have X-ray images of normal, influenza, and COVID-19 patients as a dataset. For the classification of these images, deep learning algorithms are applied, and we get three categories as a result, i.e., normal, influenza, and COVID-19 patient's images. So, the problem is classifying chest X-ray images of the discussed categories using proposed deep learning models.

## 4. Symptoms of COVID-19 and Influenza

Infectious respiratory problems are caused by influenza and COVID-19 viruses [27]. Since the symptoms of influenza and COVID-19 are mostly similar [28, 29], it may be hard to distinguish based on symptoms alone, and tests could be required to confirm a diagnosis. Fever or feeling feverish/chills, cough, shortness of breath or trouble breathing, stuffy or runny nose, muscle aches and pains, headaches, and fatigue are all typical COVID-19 and flu symptoms [30]. When an individual becomes infected with flu or COVID-19 and the onset of illness symptoms, one or more days can be passed without any feeling of illness. When somebody has COVID-19, they can be infectious for longer than when they have flu. Before showing symptoms, most people with flu are infectious for around one day [31]. Flu tends to be most infectious in older children and adults within the first 3-4 days of their illness, but many individuals are contagious for up to 7 days [32]. COVID-19 and influenza are contagious and can spread from person to person in close quarters (within about 6 feet). Both are transmitted primarily by droplets produced by sick people or by coughing, sneezing, or talking [33]. Shaking hands or touching a virus-infected surface or item and then touching their mouth, nose, or eyes could all result in infection [34]. The flu virus and the virus that causes COVID-19 can be transmitted by people who have never had symptoms or have only mild symptoms (asymptomatic). COVID-19 patients have different conditions, i.e., mild, moderate, severe, or critical, according to the nature of the disease. Mild sickness is defined as patients who exhibit any of the COVID-19 symptoms, but no breathing problem, dyspnea, or abnormal chest imaging.

During clinical examination or chest imaging, patients with moderate COVID-19 have oxygen saturation (SpO_2_) greater than or equal to 94% and show evidence of lower respiratory disease. Patients with a severe level of COVID-19 had a SpO_2_ of less than 94% on room air at sea level, a PaO_2_/FiO_2_ ratio of 300 mm Hg, and a breathing rate of more than 30 breaths/min. Patients in critical condition of COVID-19 are those who have respiratory failure, septic shock, and/or multiple organ dysfunction. Younger people have a milder COVID-19, whereas older patients have a more severe COVID-19. Moreover, it propagates with more speed from mild to critical in geriatric patients than younger patients. Patil et al. [35] presented an article on the effects of COVID-19 on different age groups. They explored several techniques for detecting COVID-19 in various age groups. According to this study, the age groups 30–44, 45–59, and 60–74 are the most affected by COVID-19. Critical COVID-19 disorders are common in those aged 60–74, resulting in a significant fatality rate in this age group. Turke [36] presented a study to discuss the various reasons for the lower severity level in younger than older patients. After the experiments found that younger people are less affected than older people because young people have an excellent immune system. Despite the many factors that influence infectious disease outcomes, it seems logical to claim that paying more attention to the shape of natural selection over time would be advantageous. Grasp how host-pathogen interactions evolve with age will necessitate a full understanding of the human lifespan.  Table 2.

## 5. Proposed System

**Figure 2:**
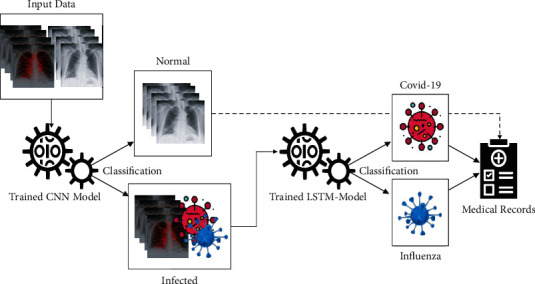
Proposed diagram.

First, we gather chest X-ray images from standard machine learning platform Kaggle, UCI, and some data from hospitals of Islamabad. These data were categorized into two categories, i,e., normal and infected. Deep learning models are trained to classify normal and infected images on these data. Normal images data were discarded, and COVID-19 and influenza images were used for the second model training. The second model classifies COVID-19 and influenza images based on the training (Figure 2).

### 5.1. CNN and LSTM Algorithms

**Figure 3:**
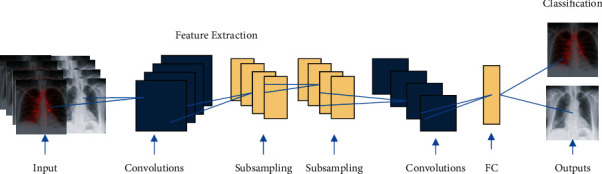
CNN architecture.

The CNN is a neural network popular and widely used in computer vision applications [37]. The CNN learns spatial hierarchies of features automatically and adaptively, and as shown in Figure 3, different layers are performed, e.g., convolution layers, pooling layers, and fully linked layers [38]. We can derive higher representations for image information using the CNN model. Unlike traditional image recognition, which encourages us to identify the image features ourselves, this method does not enable us to do so [39].

LSTM is another type of the NN that can handle long-series data and gradient vanishing problems. LSTM is better suited for time-series data with extended periods or delays time units than the CNN and RNN [40]. LSTM employs memory cells explicitly designed to retain information over long periods to capture long-term dependencies.

**Figure 4:**
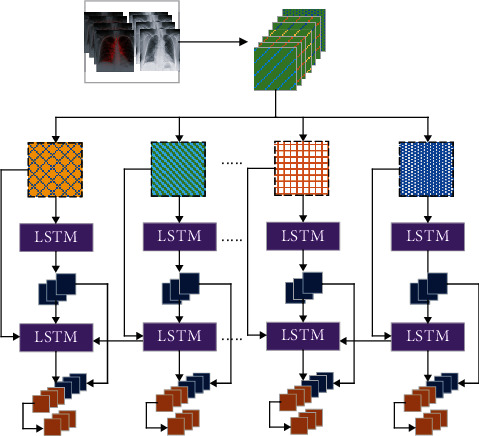
LSTM architecture.

Figure 4 shows an LSTM consisting of different functions, namely, Tanh and sigmoid, and A represents the hidden states [41].

LSTM starts with a forgotten gate, which is described as

(1) $$\begin{array}{c} F_{t}=\sigma \left(Y_{t}\times \left[h_{t},X_{t}\right]+C_{\sigma }\right). \end{array}$$

To determine the level of information being forgotten in the initial state's memories, a sigma (*σ*) feature is combined with the previously hidden layer (h_t-1_) and current feedback (*x*_o_). Then, the input gate determines the new data applied to the unit. At first, a sigmoid layer defines the values that will be altered, as follows:

(2) $$\begin{array}{c} I_{t}=\sigma \left(Y_{t}\times \left[h_{t},X_{t}\right]+C_{t}\right). \end{array}$$

After that, a tanh layer proposes a vector of new data applied to the state.

(3) $$\begin{array}{c} C_{t}=\tanh\left(Y_{\sigma }\times \left[h_{t},X_{t}\right]+C_{\sigma }\right),\\ C_{t}=F_{t}*C_{t-1}+I_{t}*C_{t}. \end{array}$$

The output gate calculates the cell's output depending on the cell state at instant “t” as well, as the most recently added data will be

(4) $$\begin{array}{c} \sigma_{t}=\sigma \left(Y_{\sigma }\times \left[h_{t-1},X_{t}\right]+C_{\sigma }\right). \end{array}$$

The memory state *σ*_t_ is the output gate that is being used to determine the memory output quantity:

(5) $$\begin{array}{c} h_{t}={\sigma_{t}}^{*}\;\tan h\left(C_{t}\right). \end{array}$$

### 5.2. Dataset Gathering

**Figure 5:**
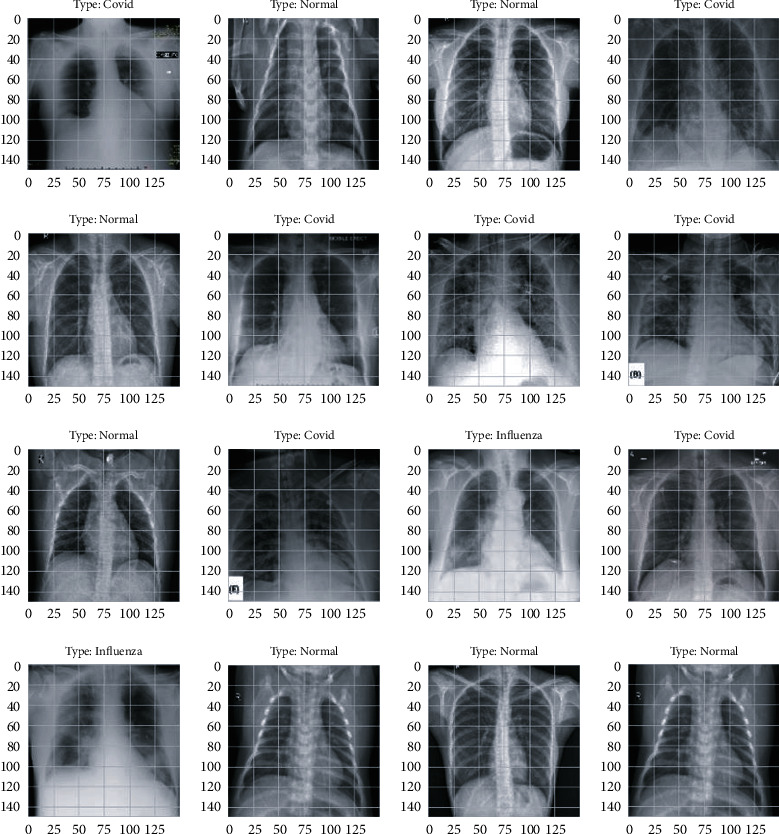
Images from dataset.

In the data gathering section, we collected datasets for the proposed problem. Images of COVID-19 and influenza were collected in this phase. The dataset was gathered from the Kaggle machine learning platform, UCI repository, and some hospitals of Islamabad. Some of the images collected in this phase are shown in Figure 5.

### 5.3. Data Preparation and Model Inputs

The proposed dataset that contains X-ray images is collected in the first stage and divided into two classes. The infected images are divided into two classes, i.e., COVID-19 and influenza. 70% of data were utilized for model training, while 30% was used for testing purposes. Because this dataset had images of varying sizes, all pictures were resized to a single dimension and rescaled to enable equal processing durations during the CNN and LSTM training experiments. A global process was established to accommodate standard customized architecture inputs that employ deep learning to automatically approximate a CNN-based and LSTM-based infection rate prediction from images (310 × 310). The scaling phase of the final customized model using the CNN is preceded by a preliminary break of the original image. The 30% images were also resized and rescaled that is used as a test set for evaluating the proposed models.

### 5.4. Model Training

The proposed deep learning models have been built that receive two sets of image classifications, the estimated probability for each class as input and output (e.g., normal case and infected case), and a set of deep learning models (CNN and LSTM) that accept two sets of image categories as input and output, the estimated probability for every class (influenza case and COVID-19 case) has also been developed during the study. These models are trained and evaluated on the datasets. In the evaluation phase, models are evaluated using various hyperparameters. The best model has been retained from the trained models.

A TensorFlow workflow is utilized for the development of proposed models. The dual-use model's prediction stage, in particular, is based on a second-level data analysis. A subimage sequence is first generated by putting a normal grid onto the original test images while entirely covering the images. The image is broken down into several image blocks, each corresponding to a grid cell. This operation expands the training set's size while minimizing image information loss. When actual data are scaled for transforming inputs into a standard DL system, this loss is frequent. The LSTM model then uses each image block to generate local predictions for estimating health indicators. Figure 2 shows the working of the CNN and LSTM model to classify COVID-19 and influenza images.

### 5.5. Model Testing

**Figure 6:**
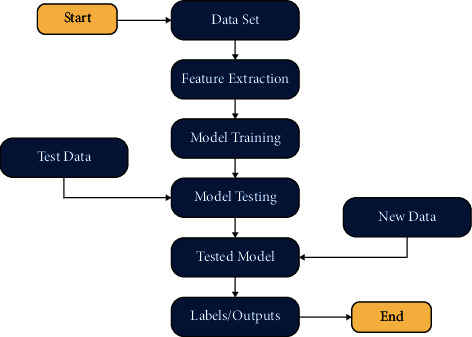
Flow diagram of the proposed model.

After the training phase, testing is performed on the trained models. This phase is done to check the accuracy of the trained model. Unlabeled data are given to the classifiers, and the classifier gives the label of the cleaned data in this phase. The testing dataset was used to evaluate the proposed models, and models performed well on the given dataset (Figure 6). The results of the study were very promising. Finally, the results were shown in graphs, so that analysis could be performed on the results.

## 6. Experiment Results

**Figure 7:**
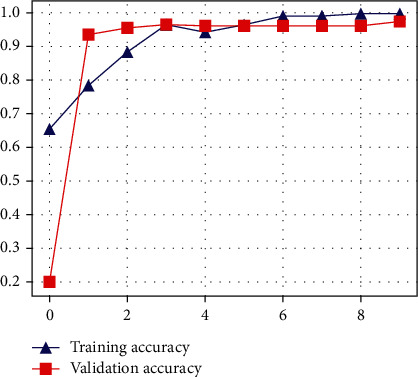
Accuracy of the proposed CNN model.

**Figure 8:**
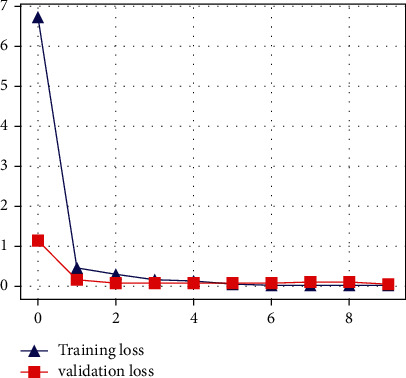
Loss score of the proposed CNN model.

**Figure 9:**
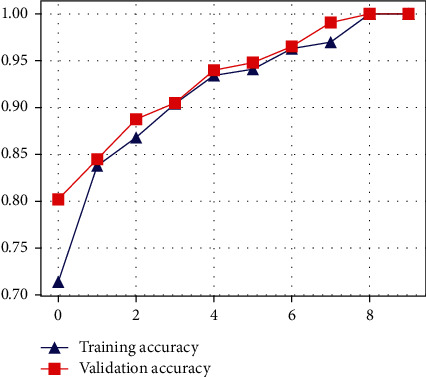
Accuracy of the proposed LSTM model.

**Figure 10:**
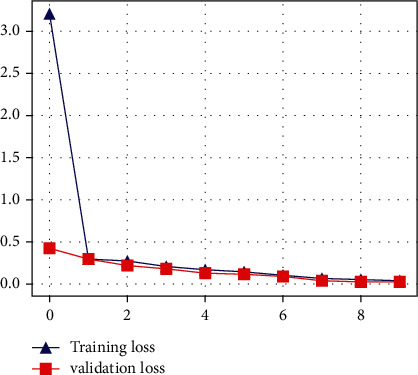
Loss score of the proposed LSTM model.

The proposed CNN model's accuracy for the classification of normal and infected images is shown in Figure 7. As shown in Figure 7, the proposed model achieved 94% accuracy. However, the proposed model got a loss score of 0.05%, as shown in Figure 8. The LSTM-based architecture is incredibly responsive to influenza and COVID-19 classification. The LSTM-based model outperforms other models with 98% accuracy and 0.34% loss value. The accuracy and loss value of the proposed LSTM model is shown in Figures 9 and 10. The results of the LSTM model demonstrate that influenza detection of COVID-infected individuals is very reliable, and viral recognition is satisfactory, given the variety of utilized radiography sources. Furthermore, our models detect influenza and COVID-19 early since the quasitotality of cases has been detected.

**Figure 11:**
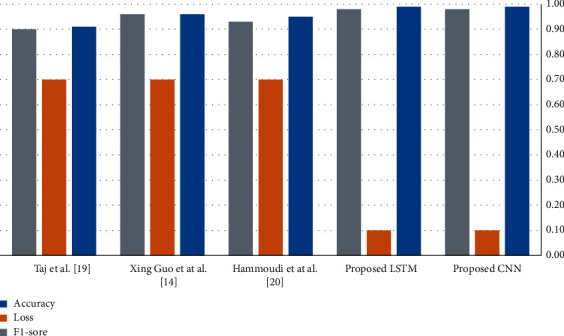
Comparison of proposed techniques.

Figure 11 shows the performance comparison of the suggested models. Figure 11 shows that the CNN model got satisfactory results, but the LSTM model outperforms the CNN model.

In addition to the conventional approach, the efficiency of the traditional techniques for classifying normal and infected images using adapted CNN-based architecture is shown in Figure 11; the traditional methods achieved low accuracy compared to our proposed models on discussed image data as shown in Figure 11 classification accuracy of 95.72%.

We employed four parameters to assess our suggested model's overall performance on four performance metrics. These metrics include accuracy, F1-score precision, and recall of developed models. The ratio of correct forecasts to total predictions is referred to as accuracy, and it is a measure of the model's correctness:

(6) $$\begin{array}{c} \text{Accuracy}=\text{TP}+\frac{\text{TN}}{\text{TP}}+\text{TN}+\text{FP}+\text{FN.} \end{array}$$

The ratio of accurately predicted positive data to entire expected positive data is known as precision:

(7) $$\begin{array}{c} \text{Precision}=\frac{\text{TP}}{\text{TP}}+\text{FP}. \end{array}$$

The ratio of the number of true positive predictions to the total number of correctly predicted outcomes is called recall, and it tests the model's sensitivity:

(8) $$\begin{array}{c} \text{Recall}=\frac{\text{TP}}{\text{TP}}+\text{FN.} \end{array}$$

The model's testing accuracy is also measured by the F1-score. The harmonic mean of accuracy and recall is determined as the F1-score.

(9) $$\begin{array}{c} \text{F}1-\text{score}=2\cdot \text{precision}\cdot \frac{\text{recall}}{\text{precision}}+\text{recall.} \end{array}$$

## 7. Conclusion

Deep learning tailored models were proposed to classify and diagnose COVID-19 and influenza. Efficient classifiers are developed to determine if an input X-ray image is normal, influenza, or COVID-19 infected. When the classification output is COVID-19, the patient is highly likely to be a true positive. As a result, a patient with influenza during an epidemic has a high likelihood of being detected by our proposed models. We employed a chest X-ray dataset, and proposed models exceeded 98% average accuracy on normal, influenza, and COVID-19 patients X-ray images.

## Figures and Tables

**Table 1:** Comparison table of related work.

Author	Dataset	Disease	Technique	Accuracy (%)
Kumar Nath et al. [1]	Chest X-ray and CT images	COVID-19	Deep learning	X-ray images: 99.68; CT images: 71.81
Henzel et al. [17]	Questionnaires	COVID-19	Machine learning	-
Bernese et al. [18]	X-ray images	COVID-19	Deep learning	96.7
Goto et al. [19]	Viruses and cells	Influenza	HA cleavage	-
Khan et al. [20]	Custom data	Influenza	Machine learning	90
Yin et al. [21]	Time-series data of influenza	Influenza	Deep learning	98-99
Guo, X., et al. [22]	Chest CT images	COVID-19 and influenza	Machine learning	96.6
Taj et al. [24]	Time series data of influenza	COVID-19	Deep learning, machine learning	-
Hammoudi et al. [25]	Chest X-ray images	COVID-19	Deep learning	95.7
Kassania et al. [13]	X-ray and CT images of chest	COVID-19	Deep learning	98
Cabitza et al. [14]	Blood test data	COVID-19	Machine learning	90
Saygili et al. [26]	X-ray and CT images of chest	COVID-19	Machine learning	98
M. Ismael and Sengur [15]	Chest X-ray images	COVID-19	Deep learning	94.7

**Table 2:** Symptoms comparison.

Symptoms	Influenzas	COVID-19	Similarities
Fever			Yes
Cough			Yes
Breathing problems			Yes
Conjunctivitis		**×**	No
Fatigue			Yes
Sore throat			Yes
Loss of taste or smell	**×**		No
Stuffy nose			Yes
Aches and pains		**×**	No
Vomiting			Yes
Diarrhoea			Yes
Rash of the skin	**×**		No
Effects on the lungs	**×**		No
Duration (estimate)	3–7 days	10–20 days	No

## Data Availability

The data used to support the findings of this study are available from the corresponding author upon request.
